# Comparison of cyanobacterial microcystin synthetase (*mcy*) *E* gene transcript levels, *mcy E* gene copies, and biomass as indicators of microcystin risk under laboratory and field conditions

**DOI:** 10.1002/mbo3.173

**Published:** 2014-05-17

**Authors:** Felexce F Ngwa, Chandra A Madramootoo, Suha Jabaji

**Affiliations:** 1 Department of Bioresource Engineering, McGill UniversityMacdonald Campus, 21111 Lakeshore Road, Ste. Anne de Bellevue, Québec, Canada, H9X 3V9; 2 Department of Plant Science, McGill UniversityMacdonald Campus, 21111 Lakeshore Road, Ste. Anne de Bellevue, Québec, Canada, H9X 3V9

**Keywords:** Cyanobacterial bloom, gene expression, *mcyE*, microcystin, reverse transcription qPCR

## Abstract

Increased incidences of mixed assemblages of microcystin-producing and nonproducing cyanobacterial strains in freshwater bodies necessitate development of reliable proxies for cyanotoxin risk assessment. Detection of microcystin biosynthetic genes in water blooms of cyanobacteria is generally indicative of the presence of potentially toxic cyanobacterial strains. Although much effort has been devoted toward elucidating the microcystin biosynthesis mechanisms in many cyanobacteria genera, little is known about the impacts of co-occurring cyanobacteria on cellular growth, *mcy* gene expression, or *mcy* gene copy distribution. The present study utilized conventional microscopy, qPCR assays, and enzyme-linked immunosorbent assay to study how competition between microcystin-producing *Microcystis aeruginosa* CPCC 299 and *Planktothrix agardhii* NIVA-CYA 126 impacts *mcyE* gene expression, *mcyE* gene copies, and microcystin concentration under controlled laboratory conditions. Furthermore, analyses of environmental water samples from the Missisquoi Bay, Quebec, enabled us to determine how the various potential toxigenic cyanobacterial biomass proxies correlated with cellular microcystin concentrations in a freshwater lake. Results from our laboratory study indicated significant downregulation of *mcyE* gene expression in mixed cultures of *M. aeruginosa* plus *P. agardhii* on most sampling days in agreement with depressed growth recorded in the mixed cultures, suggesting that interaction between the two species probably resulted in suppressed growth and *mcyE* gene expression in the mixed cultures. Furthermore, although *mcyE* gene copies and McyE transcripts were detected in all laboratory and field samples with measureable microcystin levels, only *mcyE* gene copies showed significant positive correlations (*R*^2^ > 0.7) with microcystin concentrations, while McyE transcript levels did not. These results suggest that *mcyE* gene copies are better indicators of potential risks from microcystins than McyE transcript levels or conventional biomass proxies, especially in water bodies comprising mixed assemblages of toxic and nontoxic cyanobacteria.

## Introduction

Cyanobacterial blooms often contain mixed assemblages of morphologically indistinguishable toxic and nontoxic strains from the same genus (Vezie et al. 1998) or multiple cyanobacterial genera (Vaitomaa et al. 2003; Rantala et al. 2006; AL-Tebrineh et al. 2012). *Anabaena*, *Microcystis*, *Planktothrix*, and *Nostoc* are among the most common potential microcystin-producing cyanobacteria genera in many freshwater lakes (Chorus and Bartram 1999; Sivonen and Börner 2008) including the Missisquoi Bay (Ngwa et al. 2012).

Microcystins (MCs) are among the most widespread cyanotoxins in freshwater bodies, with over 90 structural variants identified globally (Welker and Von Döhren 2006). Human and wildlife fatalities resulting from acute exposure to lethal doses of various microcystin structural variants have been reported (Jochimsen et al. 1998; Briand et al. 2003; Wood et al. 2010). Chronic exposure to sublethal doses of MCs has been shown to initiate tumor development in laboratory animals (Falconer 1991; Nishiwaki-Matsushima et al. 1992; Ito et al. 1997). Although there is no direct experimental evidence associating MCs to tumorigenesis in humans, epidemiological studies have nevertheless found correlations between incidence of MCs in drinking water and/or food, to development of certain types of cancers in China (Yu 1995; Ueno et al. 1996; Zhou et al. 2002). These studies coupled with their demonstrable tumor promoting effects in laboratory rodents have led to the classification of MCs as “possible human carcinogens (Class 2B)” by the International Agency for Research on Cancer (IARC; Grosse et al. 2006).

The global relevance of MCs and *Microcystis* has led to dedicated efforts toward elucidating the role of this toxin in cyanobacteria metabolism and the factors regulating its biosynthesis. A good number of studies have shown that nutrients (Rapala et al. 1997; Orr and Jones 1998; Vézie et al. 2002; Xu et al. 2010), temperature (Watanabe and Oishi 1985; Amé and Wunderlin 2005; Davis et al. 2009), light (Kaebernick et al. 2000; Kardinaal et al. 2007; Leblanc Renaud et al. 2011), and pH (Van Der Westhuizen and Eloff 1983; Krüger et al. 2012) affect cyanobacterial growth and microcystin production in various ways. Results from many of these studies are rather contradictory; hence the precise biological function of MCs and the factors regulating its production are still contentious.

Microcystin-producing cyanobacteria contain the microcystin synthetase (*mcy*) genes that are generally absent in their nontoxic counterparts. Thus, detection of these toxin biosynthetic genes in samples usually indicates presence of potentially toxic cyanobacteria. Genome sequencing and characterization of the microcystin synthetase gene clusters in *Anabaena* (Rouhiainen et al. 2004), *Microcystis* (Nishizawa et al. 2000; Tillett et al. 2000), and *Planktothrix* (Christiansen et al. 2003) species has elucidated the roles of various *mcy* genes in the microcystin biosynthesis pathway.

Elucidation of the molecular basis for microcystin biosynthesis, for example, has resulted in increased use of molecular techniques to discriminate toxic from nontoxic cyanobacterial blooms. A majority of molecular methods employ PCR amplification of cyanobacterial genomic DNA to either detect presence/absence of various *mcy* genes in samples as in conventional PCR (e.g., Tillett et al. 2001; Kurmayer et al. 2002; Via-Ordorika et al. 2004; Yoshida et al. 2005) or quantify concentrations of various *mcy* gene copies as in quantitative real-time PCR (e.g., Kurmayer and Kutzenberger 2003; Vaitomaa et al. 2003; Fortin et al. 2010; AL-Tebrineh et al. 2012). Although DNA-based PCR detection, differentiation, and quantification of *mcy* genes provide vital information on proportions of potential microcystin-producing cyanobacteria, it nevertheless offers little insight into active *mcy* gene transcription. Considering that deletional or insertional mutagenesis of *mcy* genes is known to produce nontoxic mutants incapable of expressing the *mcy* genes (Kaebernick et al. 2001; Christiansen et al. 2008; Ostermaier and Kurmayer 2009), it has been suggested that genomic DNA-based qPCR methods might be subject to toxigenicity overestimation in environmental samples with high proportions of such mutants.

Synthesis of MCs follows a series of steps starting with transcription of *mcy* genes into *mcy* mRNAs, translation of mRNAs into polyketide synthases (PKSs), nonribosomal peptide synthetases (NRPSs), mixed peptide synthetases, and tailoring enzymes that assemble various constituent amino acids into the microcystin structure (Misson et al. 2012). Reverse transcription quantitative real-time PCR (RT-qPCR) analysis of total cyanobacterial RNA allows for measurement of transient *mcy* mRNA transcripts, and putatively, the concentrations of potential microcystin-producing cyanobacteria which are actively transcribing the *mcy* genes at the time of sampling. It is thus assumed that knowledge of concentrations of actual microcystin producers in a given bloom could result in a more reliable assessment of the risk of exposure to this toxin.

Although RT-qPCR can provide vital information on potential bloom toxicity that cannot be obtained from current DNA-based qPCR approaches, use of this technique in monitoring toxigenic cyanobacteria is not widespread probably due to the greater lability of RNA relative to DNA, as well as inherent difficulties in extracting and preserving high-quality RNA from complex environmental samples (Sharkey et al. 2004; Gadkar and Filion 2012). Few studies on reverse transcription PCR analysis of toxigenic cyanobacteria have mostly involved qualitative measurement of presence or absence of Mcy transcripts in laboratory cultures (e.g., Kaebernick et al. 2000, 2002; Mikalsen et al. 2003) or field samples (Gobler et al. 2007). Quantitative estimation of various *Microcystis* Mcy gene transcript levels under laboratory (e.g., Schatz et al. 2007; Sevilla et al. 2008; Rueckert and Cary 2009) and field (e.g., Wood et al. 2011; Misson et al. 2012) conditions have also been successfully realized.

The paucity in knowledge on use of Mcy transcripts as proxies for toxicity estimation notwithstanding, very little has been done to comprehend the impacts of co-occurring cyanobacteria on growth and toxicity of blooms. Understanding the relationships between cell counts, Mcy transcript levels, *mcy* gene concentrations, and actual cellular microcystin concentrations could therefore lead to identification of the most reliable indicators of microcystin risk under controlled laboratory and field conditions.

In order to address the identified knowledge gaps, the present study investigated the changes in biomass, *mcyE* gene copies, McyE gene transcripts, and microcystin concentration in mono- and mixed cultures of *Microcystis aeruginosa* CPCC 299 and *Planktothrix agardhii* NIVA-CYA 126 during 21 days of growth. Supplementary analyses of water samples collected from a freshwater lake during summer 2011 enabled us to further evaluate how variation in *mcyE* gene expression, *mcyE* gene copies, or cyanobacterial biomass with microcystin production, could impact the utility of these putative proxies as indicators of microcystin risks in a freshwater body.

The *mcyE* gene was chosen over all other genes in the *mcy* cluster because of the important role it plays in the synthesis and incorporation of the toxicity determining ADDA (3-amino-9-methoxy-2, 6, 8-trimethyl-10-phenyldeca-4, 6-dienoic acid) and d-Glu (d-glutamic acid) moieties into the MC structure. Furthermore, the relatively invariant nature of these amino acids makes the *mcyE* regions reliable biomarkers for detection of microcystin-producing cyanobacteria.

## Materials and Methods

### Organisms and culture conditions

Unialgal cultures of toxic *M. aeruginosa* CPCC 299 (hereafter *M. aeruginosa*) and *P. agardhii* NIVA-CYA 126 (hereafter *P. agardhii*) were purchased from the Canadian Phycological Culture Collection (CPCC; formerly the University of Toronto Culture Collection [UTCC]) and the Norsk Institutt for Vannforskning (NIVA), respectively. The organisms were grown in 125 mL sterile BG-11 media in 250 mL Erlenmeyer flasks placed in a Conviron growth chamber maintained at a temperature of 25°C and illuminated with 12 h of white fluorescent light supplied at a photon flux density of 30 *μ*mol m^−2^ sec^−1^. Cultures were allowed to acclimatize for one week in fresh BG-11 under stipulated culture conditions prior to start of competition experiments.

### Competition experiments

Acclimatized cultures of *M. aeruginosa* and *P. agardhii* were each aseptically transferred into separate triplicate 2 L Erlenmeyer flasks containing 1 L of BG-11 for the monoculture assays. For the mixed culture experiments, *M. aeruginosa* and *P. agardhii* were added into 2 L Erlenmeyer flasks (in triplicates) containing 1 L BG-11 such that each flask contained approximately the same starting concentration (10^5^ cells mL^−1^) of each strain. All nine flasks were randomly placed in a Conviron growth chamber maintained at 25°C temperature and 12 h:12 h light:dark cycle at 30 *μ*mol photons m^−2^ sec^−1^ provided by cool white fluorescent bulbs. Each flask was aseptically sampled on 5th, 9th, 13th, 17th, and 21st November 2012 (hereafter days 5, 9, 13, 17, and 21 of the experiment) with 20 mL aliquots immediately filtered onto GF/C filter paper (Whatman, Cardiff, England), wrapped in aluminum foil and preserved at −80°C (for nucleic acid and microcystin analyses) or analyzed immediately for chlorophyll-a. Samples for microscopy were preserved in acid Lugol and kept in the dark for subsequent cell counting.

### Field samples

A total of 32 surface water samples were collected from two littoral stations VQ-B2 (45° 04′ 57″N; 73° 08′ 52″W) and PH-A (45° 02′ 22″N; 73° 04′ 46″W), located in the Missisquoi Bay, Quebec (Fig. S1), between May and November 2011. These stations were chosen because of their proximity to areas that support important economic and recreational activities (summer resorts, aquatic sports, and fisheries) and the water intake for the drinking water treatment plant of surrounding towns. Five hundred milliliter aliquots were filtered onto GF/C filters (Whatman), wrapped in aluminum foil, and preserved at −20°C for nucleic acid extraction and toxin analyses.

### Biomass measurement

Growth of *M. aeruginosa* and *P. agardhii* was assessed by measuring the chlorophyll-a and cell concentrations on days 5, 9, 13, 17, and 21 of culture experiments. Chlorophyll-a was extracted from GF/C filters using hot 90% ethanol followed by spectrophotometric analysis as described previously (Sartory and Grobbelaar 1984). Enumeration of cyanobacterial cells was carried out using a light microscope as previously described (Ngwa et al. 2012). In order to estimate *P. agardhii* cell counts, *P. agardhii* cells were assumed to be cylindrical in shape. Dimensions of at least 50 *Planktothrix* filaments (length and diameter) were measured using a SteREO Discovery.V20 microscope (Carl Zeiss Canada Ltd., Toronto, Ontario, Canada). *Planktothrix agardhii* cell numbers were estimated from total filament counts and mean filament length by assuming a mean cell length of 5 *μ*m (Suda et al. 2002; Jacquet et al. 2005).

### Nucleic acid isolation and reverse transcription

Preserved GF/C filters for DNA and RNA isolation were separately ground into powder in liquid N_2_ cooled mortars and pestles. To avoid nucleic acid degradation, samples were completely submerged in liquid N_2_ throughout the grinding process.

Total RNA was extracted from entire ground filters using RNeasy® Plant Mini kit (Qiagen Inc., Mississauga, Ontario, Canada) following manufacturer's recommendations. RNA concentration and integrity were assessed spectrophotometrically with NanoDrop® ND1000 (NanoDrop, Wilmington, DE) followed by 1.2% formaldehyde-agarose gel electrophoresis.

Prior to RT, residual gDNA coextracted with RNA was destroyed with DNase wipeout buffer. A total of 100 ng of purified RNA was reverse transcribed using the QuantiTech® reverse transcription kit (Qiagen Inc.) following the kit manufacturer's protocol. Transcribed cDNA concentration and integrity were checked with ND1000 spectrophotometer. Genomic DNA contamination of reverse transcribed cDNA was verified via conventional PCR of no reverse transcriptase (NoRT) samples with 16S rRNA, rpoC1, and genus-specific MicmcyE and PlamcyE primer pairs (Table1), followed by 1.5% agarose gel electrophoresis. Transcribed cDNA was preserved at −20°C for subsequent PCR applications.

**Table 1:** List of primers used in this study.

Target gene	Primer name	Primer sequence (5′–3′)	Melting temp (°C)	Reference
Microcystin synthetase E	MicmcyE-415F^1^	CCTGCACTCCCTGAGAGAAC	60	Ngwa et al. (2013)
	MicmcyE-581R	AATGACCGCCAA TTTCAA AG	60	
Microcystin synthetase E	PlamcyE-427F^1^	GATTGCACTCAA TGAAACCG	60	Ngwa et al. (2013)
	PlamcyE-610R	AACGTGGGTTACGATTCTCG	60	
16S ribosomal RNA	16srRNA-RTF^2^	CTGAAGATGGGCTCGCGT	61.7	Imamura et al. (2006)
	16srRNA-RTR	CGTATTACCGCGGCTGCT	61.3	
RNA polymerase *γ* subunit	rpoC1F^2^	CCTCAGCGAAGATCAATGGT	60.2	Alexova et al. (2011)
	rpoC1R	CCGTTTTTGCCCCTTACTTT	60.3	

1 The MicmcyE and PlamcyE primer pairs are specific for the *Microcystis* and *Planktothrix mcyE* genes.

2 The 16S rRNA and rpoC1 primer pairs are reference genes used to normalize the mcyE gene expression data.

Genomic DNA was extracted from ground filters with the DNeasy® Mini Plant kit (Qiagen Inc.) as per the kit manufacturer's instructions. DNA quality and quantity were assessed with ND1000 spectrophotometer (NanoDrop), followed by electrophoresis on 1.5% agarose gel. Purified DNA was preserved at −20°C until further analysis.

### Primer design

Genus-specific primer pairs targeting the microcystin synthetase gene in *Microcystis* and *Planktothrix* genera designed with Primer 3 (Rosen and Skaletsky 2000) were tested for the specificity to respective genera as described in a previous study (Ngwa et al. 2013). Primer pairs for the16S rRNA (Imamura et al. 2006) and rpoC1 (Alexova et al. 2011) reference genes were selected from literature and tested via conventional PCR of cDNA to ensure that they amplified only the target genetic locus.

### Quantitative PCR determination of *mcyE* gene copies

The concentrations of *Microcystis* and *Planktothrix mcyE* copies were determined by quantitative PCR of gDNA, using standard curves generated from *Microcystis* and *Planktothrix mcyE* plasmid DNA as described previously (Ngwa et al. 2013). All samples were amplified in duplicate using the Mx3005P qPCR System (Agilent Technologies, Inc., Santa Clara, CA) and QuantiFast® SYBR® Green master mix (Qiagen Inc.) as per the manufacturers' recommendations. Amplifications were performed in 25 *μ*L reaction mixtures containing 300 and 200 nmol/L of forward and reverse primers for *Microcystis* or *Planktothrix mcyE* gene, respectively, 1× QuantiFast® SYBR® Green I master mix (with ROX dye), and 2 *μ*L gDNA template (10^2^ to 10^4^ fold dilutions). The PCR thermal cycle consisted of a hot start cycle at 95°C for 5 min, followed by 40 cycles comprising denaturation at 95°C for 10 sec, and a combined annealing/extension step at 60°C for 30 sec. All fluorescence data were collected at 60°C and C_T_ values determined using MxPro qPCR software v4.10 (Agilent Technologies, Inc.).

### Analysis of *mcyE* gene expression by RT-qPCR

Microcystin synthetase E (*mcyE*) gene transcript levels under laboratory and field conditions were estimated by the relative quantification approach described by Zhao and Fernald (2005). The PCR assays were performed on two target genes (*Microcystis* and *Planktothrix mcyE*) and reference genes (16S rRNA and rpoC1) using the primer pairs shown in Table1. The *Microcystis* and *Planktothrix mcyE* assays were performed as described above using 2 *μ*L (500 ng) of cDNA template instead of gDNA in each 25 *μ*L PCR reaction mixture. Amplification of reference genes on the other hand was performed in 25 *μ*L reactions each containing either 300 nmol/L of each 16S rRNA primer or 500 nmol/L rpoC1 forward and reverse primers, 1× QuantiFast® SYBR® Green I master mix, and 500 ng cDNA template. All RT-qPCR amplifications were performed using a thermal program consisting of a hot start cycle at 95°C for 5 min, followed by 40 cycles comprising denaturation at 95°C for 10 sec, and a combined annealing/extension step at 60°C for 30 sec. Melt curve analyses of data from all qPCR and RT-qPCR assays were performed as previously described (Ngwa et al. 2012).

### Quantification of gene expression

In order to quantify gene expression, raw fluorescent data were imported from the Stratagene Mx3005P qPCR machine into Miner software v4.0 (Zhao and Fernald 2005) and various software algorithms utilized to calculate C_T_ values and efficiencies based on kinetics of individual PCR reactions. The resulting C_T_ values and efficiencies were subsequently inputted into equation (1) to calculate relative expression of target and references genes as described by Zhao and Fernald (2005).

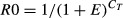
1where, *R*0 is initial template concentration, *E* is the efficiency of the PCR reaction, *C*_*T*_ represents the PCR cycle at which fluorescence significantly exceeds the background fluorescence.

In order to correct target gene expression data for differences in RNA quality and reverse transcription efficiency between samples, the relative expression of *Microcystis* or *Planktothrix mcyE* genes in various samples was normalized against expression of 16S rRNA and rpoC1 genes in the respective samples.

### Microcystin analysis

Preserved frozen filters were placed in 2 mL microcentrifuge tubes, allowed to thaw at room temperature for 5 min and rapidly subjected to two freeze-thaw cycles (liquid N_2_ for 1 min, 37°C water bath for 5 min). Samples were subsequently extracted in 75% (v/v) aqueous methanol following the procedure described in a previous study (Spoof et al. 2003). In order to prevent methanol interference with downstream enzyme-linked immunosorbent assay quantification of microcystin (Metcalf et al. 2000), all extracts were diluted with double distilled water to bring methanol concentration below 0.5% (v/v) as suggested by Rivasseau et al. (1999).

Microcystin concentration of each sample was measured in duplicate using an Envirogard® microcystin plate kit (Modern Water Inc., New Castle, DE) and a Synergy H4 Hybrid Multi-Mode microplate reader (BioTek, Winooski, VT) following the manufacturers' procedures.

### Statistical analysis

All statistical analyses were performed with SAS software package (v9.3; SAS Institute Inc., Cary, NC). Normality of data was checked using the Shapiro–Wilk normality test at the 5% significance level. Nonconforming data were log transformed to meet the normality condition and a mixed model procedure using the restricted maximum likelihood (REML) variance estimation method was implemented to detect any significant differences in cyanobacterial growth, *mcyE* copies, *mcyE* gene expression, and microcystin concentration under mono- and mixed culture treatments.

## Results

### Variation in cyanobacterial biomass

**Figure 1:**
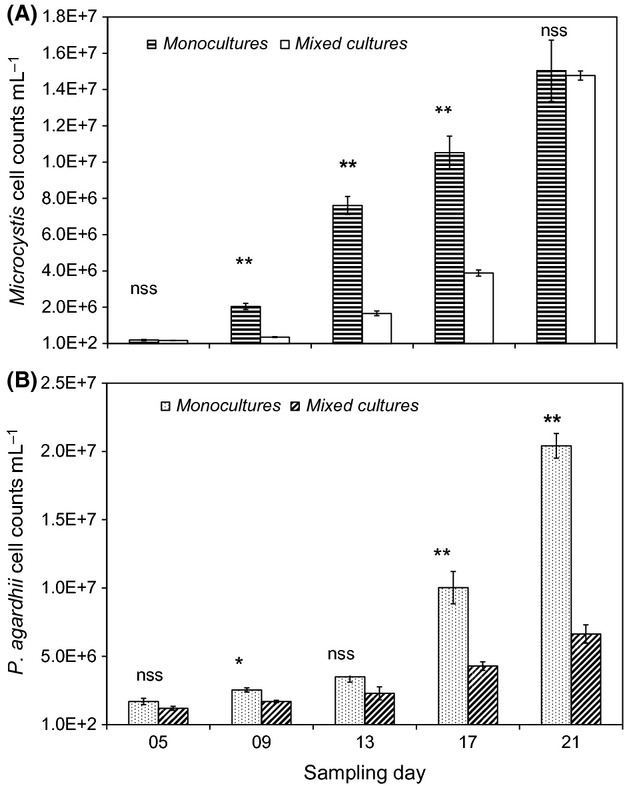
Changes *in Microcystis aeruginosa* (A) and *Planktothrix agardhii* (B) cell counts in mono- and mixed cultures. **P < 0.01; *P < 0.05; nss, not statistically significant.

The cell counts for *M. aeruginosa* and *P. agardhii* under mono- and mixed culture conditions are shown in Figure1. *Microcystis aeruginosa* cell counts in monocultures varied from 1.91 (±0.34) × 10^5^ cells mL^−1^ on day 5 to 1.50 (±0.17) × 10^7^ cells mL^−1^ on day 21 and from 1.63 (±0.50) × 10^5^ cells mL^−1^ to 1.48 (±0.03) × 10^7^cells mL^−1^ on days 5 to 21, respectively, in mixed cult-ures. *Microcystis aeruginosa* cell counts were consistently higher in monoculture than mixed cultures, although the differences were significant only on days 9, 13, and 17 (Fig.1A). *Planktothrix agardhii* cell counts in mono- and mixed culture treatments ranged between 1.70 (±0.23) × 10^6^ to 2.04 (±0.0.09) × 10^7^ cells mL^−1^ and 1.20 (±0.15) × 10^6^ to 6.64 (±0.68) × 10^6^ cells mL^−1^, respectively, on the first and last sampling days. Except for sampling day 5, *P. agardhii* counts were mostly higher in monocultures than the mixed cultures (Fig.1B). The difference in counts was, however, statistically significant only on day 21. Cyanobacterial biomass measured in terms of extractive chlorophyll-a concentration (Fig. S2) showed similar trends to cell counts determined by microscopy.

### qPCR quantification of *mcyE* copies

All primers used in this study successfully amplified a single product within the expected size range with gDNA from the respective target organisms, but gave no amplicons with nontarget organisms and negative controls. Melt curve analyses of quantitative PCR fluorescence data showed that each primer pair generated only one product with a specific melting temperature that was uniform across all samples containing target nucleic acids. Negative controls containing no template did not produce any peaks and all fluorescence data generated from such samples was always below the detection threshold. The standard curves used to estimate *Microcystis* and *Planktothrix mcyE* copies were highly linear (*R*^2^ of 0.999 for *Microcystis* and 0.998 for *Planktothrix mcyE* standards) over seven orders of magnitude.

**Figure 2:**
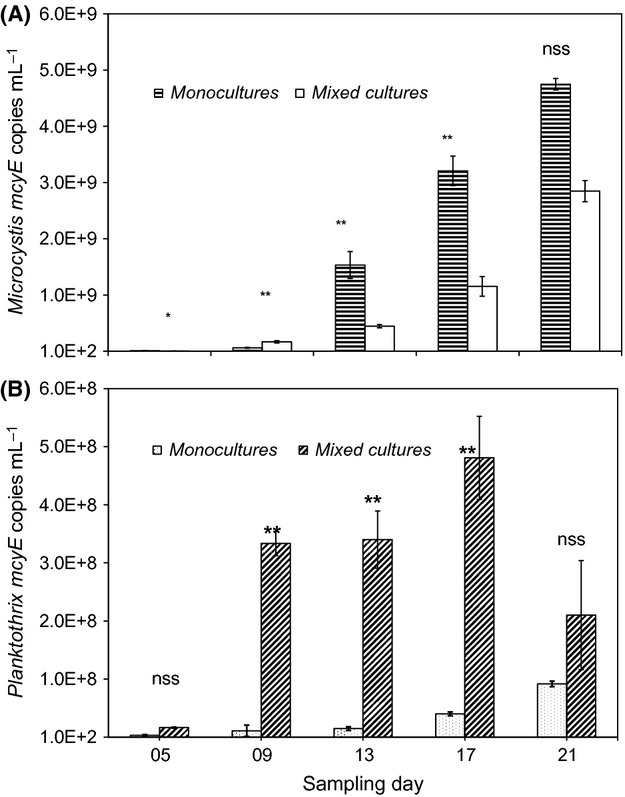
Variation in *Microcystis* (A) *and Planktothrix* (B) *mcyE* copies under mono- and mixed culture conditions. **P < 0.01; *P < 0.05; nss, not statistically significant.

*Microcystis* and *Planktothrix mcyE* copies in the mono- and mixed culture treatments were determined on gDNA extracted from samples collected on days 5, 9, 13, 17, and 21 of the culture experiments. *Microcystis mcyE* copies witnessed a steady increase from day 5 to 21 in both treatments, with values ranging from 9.41 (±3.25) ×10^6^ copies mL^−1^ to 4.75 (±0.10) × 10^9^ copies mL^−1^ in *M. aeruginosa* monocultures and 4.32 (±0.33) × 10^6^ copies mL^−1^ to 2.85 (±0.10) × 10^9^ copies mL^−1^ in mixed *M. aeruginosa* plus *P. agardhii* cultures (hereafter mixed culture; Fig.2). *Microcystis mcyE* copies showed significant treatment-by-day interaction (*P* < 0.0001). The number of *Microcystis mcyE* copies was higher in monocultures than mixed cultures on most sampling days, but for day 9 when mixed cultures registered higher *Microcystis mcyE* copies than monocultures. The differences in *Microcystis mcyE* copies between mono- and mixed cultures were statistically significant on all but the last sampling date (Fig.2A). *Planktothrix mcyE* copies, like *Microcystis mcyE* copies, showed significant treatment-by-day interaction (*P* < 0.05). Whereas *Planktothrix mcyE* copies in monoculture *P. agardhii* showed a similar trend to *Microcystis mcyE* copies, rising from 3.11 (±1.06) × 10^6^ copies mL^−1^ on day 5 to 9.15 (±0.48) × 10^7^ copies mL^−1^ on day 21, *mcyE* copies in mixed cultures, on the contrary, increased from 1.64 (±0.12) × 10^7^ copies mL^−1^ on day 5 to a maximum of 4.81 (±0.72) × 10^8^ copies mL^−1^ on day 17 before dropping to 2.10 (±0.94) × 10^8^ copies mL^−1^ by day 21 (Fig.2B). Although *Planktothrix mcyE* copies were relatively higher in the mixed than monocultures on each sampling date, the difference was significant only on days 9, 13, and 17 (Fig.2B).

### Quantification of *mcyE* gene expression

**Figure 3:**
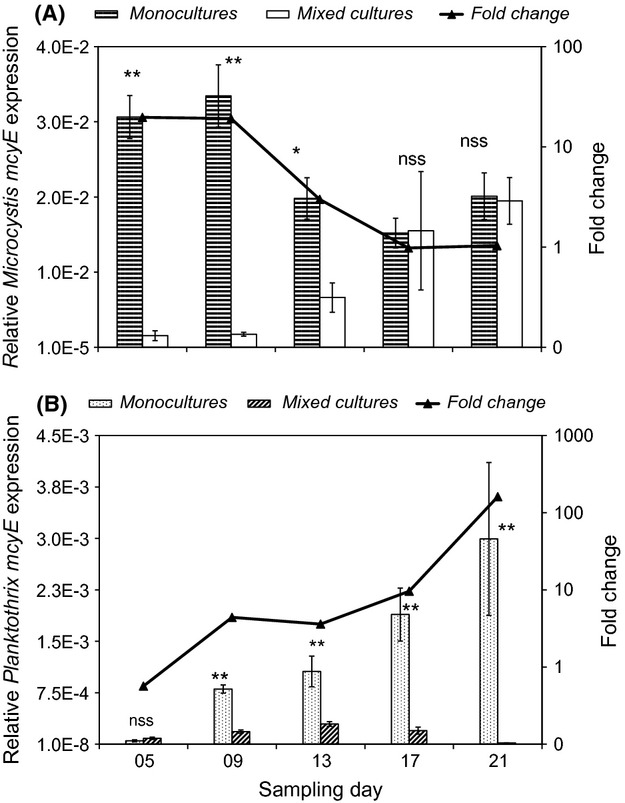
Relative expression (histograms) and fold change (triangles) of the *Microcystis* (A) and *Planktothrix* (B) *mcyE* gene under mono- and mixed culture conditions. Notice the different in scales of the *y*-axis for the *Planktothrix* and *Microcystis* graphs. Normalization of *mcyE* gene expression was performed using the 16S ribosomal RNA and RNA polymerase *γ* (rpoC1) subunit reference genes. **P < 0.01; *P < 0.05; nss, not statistically significant.

Only cDNA from target organisms was successfully amplified using the respective *Microcystis* and *Planktothrix mcyE* primer pairs, while negative controls, NoRT samples, and cDNA from nontarget organisms produced not amplicons. Expression of *mcyE* genes in *M. aerug-inosa* and *P. agardhii* from the laboratory cultures showed significant treatment-by-day interaction effects (*P* < 0.0001). *Microcystis mcyE* expression was 19.7-fold higher in monocultures than the mixed cultures on day 5, decreasing slightly to 19.2-fold on day 9 before falling steeply to equivalent values on days 17 and 21 (Fig.3A). While *Microcystis mcyE* expression in mixed cultures witnessed a steady increase from day 5 to 21, *mcyE* expression in monoculture increased from day 5 to 13, and then decreased to day 17 before increasing again on day 21. Expression of *Planktothrix mcyE* genes in monocultures, on the contrary, witnessed a steady increase from day 5 to 21, whereas in mixed cultures there was a gradual increase from day 5 to 13 and then a drop through days 17 to 21 (Fig.3B). Expression of *Planktothrix mcyE* in monocultures was significantly higher than the corresponding mixed cultures on all sampling days, but for day 5 when there was no significant difference between the two treatments. The highest fold change in *Planktothrix mcyE* expression between the two treatments was observed on day 21 when *mcyE* expression was 161-fold higher in monoculture than the mixed culture, whereas the lowest fold change was on day 5.

**Figure 4:**
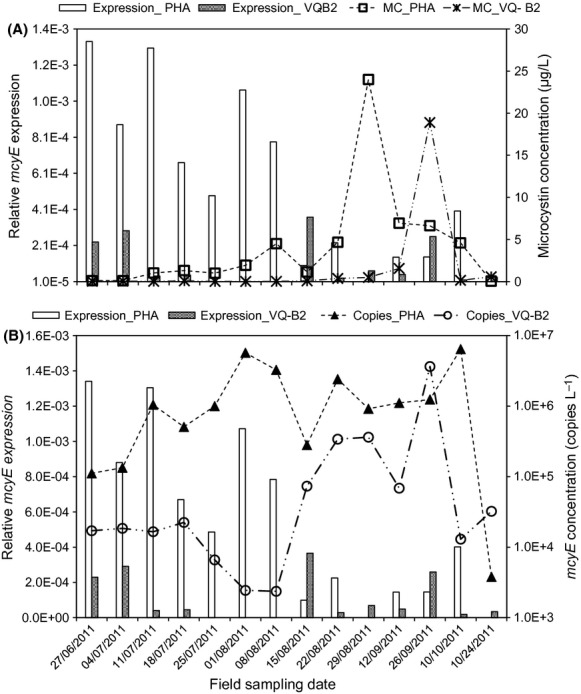
Comparison of *Microcystis mcyE* gene expression versus microcystin (A) and *mcyE* copies (B) in Missisquoi Bay samples of summer 2011. Normalization of *mcyE* gene expression was performed using the 16S ribosomal RNA and RNA polymerase *γ* (rpoC1) subunit reference genes.

Twenty-three of the 32 environmental water samples which produced RNA of quality suitable for RT-PCR were analyzed for *Microcystis* and *Planktothrix mcyE* gene expression. *Microcystis mcyE* gene was expressed in all samples analyzed but no sample produced detectable *Planktothrix* McyE gene transcript levels. Expression of *Microcystis mcyE* genes was generally higher in samples collected from station PH-A than station VQ-B2 samples collected on the same day, except for samples of 15 August and 26 September, which showed higher *mcyE* expression in station VQ-B2 than PH-A samples (Fig.4). *Microcystis mcyE* gene expression did not show any association with *Microcystis mcyE copies* (Fig.4B) determined in a previous study (Ngwa et al. 2013).

### Microcystin analysis

**Figure 5:**
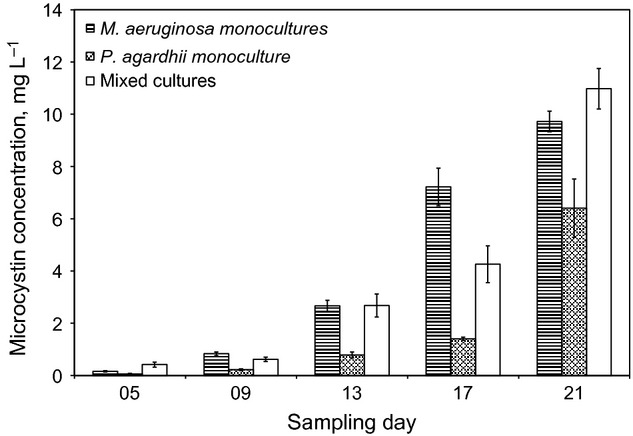
Evolution of microcystin concentration in mono- and mixed cultures of *Microcystis aeruginosa* and *Planktothrix agardhii*.

Intracellular MC concentration in monoculture *M. aeruginosa* witnessed a steady increase from 0.16 ± 0.03 mg L^−1^ on day 5 to 9.72 ± 0.39 mg L^−1^ on day 21 of the culture experiments (Fig.5). In a similar manner, monoculture *P. agardhii* had intracellular microcystin concentrations ranging from 0.06 ± 0.03 mg L^−1^ to 6.41 ± 1.11 mg L^−1^ on days 5 to 21, respectively (Fig.5). For the mixed cultures, total intracellular microcystin concentration was 0.42 ± 0.1 mg L^−1^ on day 5, increasing by more than 26 folds to 10.98 ± 0.78 mg L^−1^ on day 21 (Fig.5). While MC concentrations in monoculture *P. agardhii* were significantly lower than concentrations in mixed cultures on all but one sample day (day 21), MC concentrations in monoculture *M. aeruginosa* and mix cultures were not significantly different from each other on three of five sampling days. Microcystin concentration in the field samples ranged 0.01 to 24.0 *μ*g L^−1^ (Fig.4A) as reported in a previous study (Ngwa et al. 2013).

### Correlation analyses

In order to determine the strength of associations between *mcyE* gene expression and copies, microcystin, and various biomass proxies, Pearson correlation coefficients were calculated using log_10_ transformed data. The results of these analyses for *M. aeruginosa* and *P. agardhii* are presented in Tables2 and 3, respectively.

**Table 2:** Correlations between *Microcystis* cell counts, *mcyE* gene expression and copies, and microcystin concentration data from mono- and mixed culture experiments.

	Microcystin	Chl-a	*Microcystis mcyE* copies	*mcyE* expression	*Microcystis* cell counts
Microcystin	1.00				
Chl-a	0.96**	1.00			
*Microcystis mcyE* copies	0.94**	0.90**	1.00		
*mcyE* expression	0.29^nss^	0.38^*^	0.35^*^	1.00	
*Microcystis* cell counts	0.94**	0.93**	0.93**	0.54**	1.00

Cell counts and *mcyE* copies are expressed per mL while chlorophyll-a and microcystin are expressed as *μ*g L^−1^.

** *P* < 0.01; ^*^P < 0.05; nss, not statistically significant.

**Table 3:** Correlation between *Planktothrix* cell counts, *mcyE* gene expression and copies, and microcystin concentration data from mono- and mixed culture experiments.

	Microcystin	Chl-a	*Planktothrix mcyE* copies	*mcyE* expression	*Planktothrix* cell counts
Microcystin	1.00				
Chl-a	0.94**	1.00			
*Planktothrix mcyE* copies	0.71**	0.68**	1.00		
*mcyE* expression	0.10^nss^	0.04^nss^	0.09^nss^	1.00	
*Planktothrix* cell counts	0.79**	0.84**	0.42^*^	0.39^*^	1.00

Cell counts and *mcyE* copies are expressed per mL while chlorophyll and microcystin are expressed as *μ*g L^−1^.

** *P* < 0.01; ^*^P < 0.05; nss, not statistically significant.

Microcystin concentration correlated significantly (*r* > 0.7, *P* < 0.01) with *mcyE* copies, chlorophyll-a, and cell counts, but not with *mcyE* gene expression for both *M. aeruginosa* and *P. agardhii* (Tables2 and 3). Expression of *mcyE* in *M. aeruginosa* showed significant but weak positive association with *mcyE* copies, chlorophyll-a, or cell counts, whereas *Planktothrix mcyE* gene expression on the contrary correlated only weakly with cell counts. The association of *Microcystis mcyE* copies with various biomass proxies had Pearson correlation (*r*) values ranging from 0.82 to 0.96 (Table2), whereas that between *Planktothrix mcyE* copies and biomass variables ranged from 0.55 to 0.94 (Table3).

**Figure 6:**
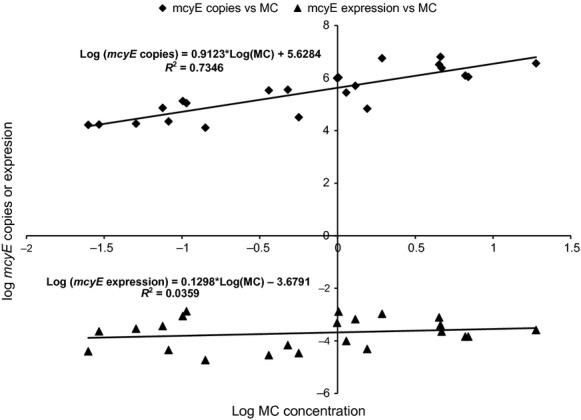
Regression of *Microcystis mcyE* gene expression and copies recorded in environmental samples against microcystin concentration.

Microcystin concentration in field samples showed significant positive association (*r* = 0.85) with the concentration of *Microcystis mcyE* copies but not McyE transcripts (Fig.6).

## Discussion

This study showed that the concentrations of the cyanobacterial cells, *mcyE* copies, MC, and McyE transcript levels in laboratory cultures were strongly dependent not only on the presence or absence of competing cyanobacteria, but also the sampling day. That is, there were significant treatment-by-day interaction effects (Table S1).

### Changes in cell counts and *mcyE* gene copies

Mixed cultures generally contained lower cell counts and *mcyE* copies than the corresponding mixed cultures, with *Microcystis mcyE* gene copies being at least two- to threefold higher in mono- than mixed cultures on most sampling days. *Planktothrix mcyE* copies were, on the contrary, between 2- and 31-fold higher in mixed cultures than in monoculture in stark contrast to the relatively lower *P. agardhii* cell concentrations in the mixed cultures. The anomalous behavior of the *Planktothrix mcyE* gene in mixed cultures is not entirely understood at the present moment and will form the basis for future research.

The lower *Microcystis* and *Planktothrix* cell concentrations recorded in mixed cultures nevertheless suggests that competitive interaction between *M. aeruginosa* and *P. agardhii* resulted in mutual growth inhibition observed in the mixed cultures. Although not directly comparable to the present study, competition experiments between toxic and nontoxic *Microcystis* have indeed shown severe inhibition of growth of nontoxic strains by the toxic strain (Schatz et al. 2005). Further analyses, however, led these researchers to suggest that other secondary metabolites (e.g., micropeptin, microviridin, or microgenin), but not MCs, were probably responsible for the growth inhibition. Other studies have, however, associated the antiagal properties of toxic *Microcystis* spp. to the release of MCs (Singh et al. 2001) or other unknown allelopathic compounds (Sukenik et al. 2002) into the surrounding media. A recent study (Mendes et al. 2012) showed that allelochemicals from mixed cultures of *Cylindrospermopsis raciborskii* and *M. aeruginosa* caused growth inhibition in different strains of *M. aeruginosa*. The fact that the *P. agardhii* strain used in this study, like *M. aeruginosa*, is also known to produce a wide array of secondary metabolites including aeruginosides, anabaenopeptins, cyanopeptolins, and microviridins (Philmus et al. 2008) which exhibit toxicity in *Daphnia* sp. (Rohrlack et al. 2004) led us to hypothesize that that growth depression witnessed in the mixed cultures might have emanated from release of growth inhibiting compounds by the competing *M. aeruginosa* and *P. agardhii*.

### Expression of microcystin synthetase (*mcy*) *E* genes

Expression of the *Microcystis* and *Planktothrix mcyE* genes was significantly downregulated in the mixed cultures relative to the respective monocultures, with up to 161-fold change for *Planktothrix mcyE* and 20-fold for *Microcystis mcyE* gene expression. While *mcyE* genes in monoculture *P. agardhii* were continuously upregulated from days 5 through 21, *mcyE* genes in *M. aeruginosa* monocultures were upregulated from day 5 to day 9 and then downregulated to levels lower than that of day 5, by days 17 and 21. Although *Microcystis mcyE* expression levels in this study were relatively lower than those reported elsewhere (Rueckert and Cary 2009), the up- or downregulated patterns were rather similar. According to Rueckert and Cary (2009), *Microcystis mcyE* was significantly upregulated to a peak by the mid-log phase (day 8 to 12) before being downregulated to almost insignificant levels by day 38 of the experiment. Unfortunately, these researchers did not provide microcystin concentration data to corroborate the observed gene expression patterns. The microcystin concentrations recorded in the monoculture *M. aeruginosa* did not seem to corroborate our *mcyE* expression pattern as MC concentration increased continuously from day 5 to day 21 while *mcyE* expression witnessed periodic peaks and dips during this period. While the *mcyE* expression trend in monoculture *P. agardhii* were in agreement with the increased cellular MC content from day 5 to 21, *mcyE* expression patterns in mixed culture *P. agardhii* were in discord with the observed continuous increase in MC.

Examination of the environmental data also showed substantial up- and downregulation of *mcyE* gene expression over the sampling period, with PH-A samples generally recording higher expression than the corresponding VQ-B2 samples. As with laboratory samples, *mcyE* expression patterns did not always match toxin concentration data (Fig.5A). The 27 June sample at station PH-A, for example, recorded the highest McyE transcript levels but relatively low *mcyE* copies (1.11 × 10^5^ copies L^−1^) and MC concentration (0.1 *μ*g L^−1^), whereas the 26 September sample at VQ-B2, with the highest MC concentration (18.9 *μ*g L^−1^) also had relatively high *mcyE* copies (3.64 × 10^6^ copies L^−1^) but extremely low McyE transcript levels.

The poor correlation between *mcyE* gene expression and microcystin concentration reported in the present laboratory and field studies was not supported by some previous studies. Schatz et al. (2007), for example, reported simultaneous increases in MC and McyB transcripts levels when laboratory cultures of *M. aeruginosa* KLL MG were exposed to cell-free extracts from *M. aeruginosa* cultures. Similarly, Sevilla et al. (2008) reported correlative increases in *mcyD* transcription and microcystin-LR levels in *M. aeruginosa* PCC7806 grown under iron-depleted conditions. Another laboratory evaluation of the response of *Planktothrix* sp. to changing light intensities found positive correlations between *mcyA* transcription rates and the total microcystin production rate in *P. agardhii* (Tonk et al. 2005). Kaebernick et al. (2000), on the contrary, showed transcription of *Microcystis mcyB* and *mcyD* genes to be light dependent but found no correlation between increasing *mcyB* or *mcyD* transcript levels and cellular toxin concentration, which they attributed to possible transport of microcystin out of the cell at high (68 *μ*mol of photons m^−2^ sec^−1^) light intensities.

At the field level, Gobler et al. (2007) reported somewhat consistent patterns in *mcyE* expression and microcystin levels in samples from Lake Agawam, USA, but also noted peculiar instances when MCs were very high despite nondetection of McyE gene transcripts. Wood et al. (2011) reported significant up- and downregulation of *mcyE* gene expression over short periods of time (2–5 h), but only detected McyE transcripts in 29% of the samples from Lake Rotorua, New Zealand, that had detectable levels of *mcyE* copies and MCs. Reports of continuous MC production by declining cultures of *M. aeruginosa* held in the dark (Lyck 2004), nevertheless, suggest that microcystin production can proceed even under conditions known to downregulate *mcy* gene expression.

The inconsistencies in *mcyE* copies, McyE transcript levels, and microcystin concentration reported in our study are conceivable when we consider the complexity of the cascade of biosynthetic steps involved in microcystin biosynthesis. In order to produce a microcystin molecule, genetic information must be dedicatedly transcribed from ten (in *Microcystis* spp.) or nine (in *Planktothrix* spp.) *mcy* genes into various *mcy* mRNA transcripts (measured in RT-qPCR), before translation into the toxin molecule. Consequently the final concentration of MCs in a given cell might be independent of ribosome or mRNA concentrations (Rueckert and Cary 2009), but could rather be influenced by posttranscriptional regulation of any of the ten or nine Mcy proteins catalyzing the various steps in toxin biosynthesis as well as posttranslational modifications of MC precursors. A previous study suggested that posttranslational modification of NRPSs and PKSs via dedicated phosphopantetheinyl transferase-mediated linkages might indeed be critical to the catalytic activity of NRPSs and PKSs (Walsh et al. 1997). Results from the present study therefore lead us to posit that posttranscriptional and/or posttranslational regulation of Mcy proteins or MC precursors might have resulted in the mismatch between measured cellular microcystin concentrations and McyE transcript levels. Additionally, these discrepancies can as well be attributed to conceivable temporal decoupling between *mcyE* transcription and presence of MCs in the cells as suggested by Gobler et al. (2007). The fact that most prokaryotic mRNA are transient molecules that are continuously degraded during translation (Voet and Voet 2004; Rueckert and Cary 2009), led us to theorize that the signals responsible for particularly high or low levels of MCs could have occurred well in advance of our sampling time/date.

### Correlation of analysis

Exploration of the correlations between MCs and *mcyE* copies and McyE transcripts, or various biomass variables revealed that although MCs were detected in all samples where *mcyE* genes were actively transcribed, McyE transcript levels were the poorest indicators of microcystin concentrations in both laboratory and environmental samples. While chl-a concentration was the best predictors of microcystin concentration in laboratory cultures, followed closely by *mcyE* copies and microscopic cell counts, *mcyE* copies were, on the contrary, the best indicators of microcystin concentrations in environmental water samples. The better association of MC concentration with chl-a over *mcyE* copies recorded in laboratory samples was expected considering that correlations of *Microcystis* or *Planktothrix mcyE* copies against total MCs in mixed cultures did not account for the microcystin resulting from either *P. agardhii* or *M. aeruginosa*, respectively, whereas the correlation of chl-a against MCs accounted for these differences, as both related total cyanobacterial biomass to total microcystin concentration. The reverse was true in environmental samples where *mcyE* copies (a measure of potentially toxic *Microcystis*) had better correlations with microcystin concentrations, whereas microscopic cell counts and chl-a, which measured total *Microcystis* (toxic and nontoxic) and phytoplankton biomass performed rather poorly.

We therefore submit that although chl-a, like *mcyE* gene copies, can be equally good indicators of microcystin levels in samples with high proportion of microcystin-producing cyanobacteria (as was the case with our laboratory cultures), *mcyE* gene copies are preferable proxies in environmental samples dominated by nontoxic cyanobacteria and/or green algae.

An important caveat to consider when interpreting the McyE transcript levels reported in this study is that the RNA used in the gene expression studies was extracted from cultures or field samples containing cyanobacterial cells at various growth phases; hence the mismatch between McyE transcript levels and MC concentration might have emanated from degradation of McyE transcripts in aging cells that were still producing and releasing MCs.

## Conclusions

Results from this study showed the biomass of microcystin-producing *M. aeruginosa* CPCC 299 and *P. agardhii* NIVA-CYA 126 to be significantly lower in the mixed cultures compared to the monocultures on most sampling days, suggesting plausible competitive inhibition of growth in the mixed culture media due to interaction between *M. aeruginosa* and *P. agardhii*.

Furthermore, *mcyE* gene copies and McyE transcripts in the cyanobacterial strains were affected not only by presence of competing cyanobacteria in growth media but also by the sampling day. That is, there were significant treatment-by-day interaction effects. *Microcystis* or *Planktothrix mcyE* gene expression was generally downregulated in mixed cultures relative to monocultures, in agreement with the lower biomass recorded in the mixed cultures. Although both McyE transcripts and *mcyE* copies were detected in all samples containing measurable MCs, only *mcyE* copies showed significant positive associations with cellular microcystin concentrations.

The strong associations between *mcyE* copies and MCs under laboratory (Pearson *r* of 0.94 and 0.71 for *Microcystis* and *Planktothrix mcyE*, respectively) and field (*r* > 0.80 for *Microcystis mcyE*) conditions further affirmed the superiority of genomic DNA-based qPCR measurement of *mcyE* gene concentrations compared to McyE transcripts and conventional biomass proxies as indicators of potential risks from MCs, especially in freshwater blooms comprising mixed assemblages of toxic and nontoxic cyanobacteria.

## Supporting Information

Figure S1 Location of the PH-A and VQ-B2 sampling stations in the Missisquoi Bay Quebec (*Source*: Ngwa et al. 2013).

Figure S2 Changes in chlorophyll-a concentration in *Microcystis aeruginosa* and *Planktothrix agardhii* under laboratory conditions.

Table S1 *F*-statistics for type III tests of fixed effects.

## References

[b1] Alexova R, Fujii M, Birch D, Cheng J, Waite TD, Ferrari BC (2011). Iron uptake and toxin synthesis in the bloom-forming *Microcystis aeruginosa* under iron limitation. Environ. Microbiol.

[b2] AL-Tebrineh J, Merrick C, Ryan D, Humpage A, Bowling L, Neilan BA (2012). Community composition, toxigenicity, and environmental conditions during a cyanobacterial bloom occurring along 1,100 kilometers of the Murray River. Appl. Environ. Microbiol.

[b3] Amé M, Wunderlin D (2005). Effects of iron, ammonium and temperature on microcystin content by a natural concentrated *Microcystis aeruginosa* population. Water Air Soil Pollut.

[b4] Briand J-F, Jacquet S, Bernard C, Humbert J-F (2003). Health hazards for terrestrial vertebrates from toxic cyanobacteria in surface water ecosystems. Vet. Res.

[b5] Chorus I, Bartram J (1999). Toxic cyanobacteria in water.

[b6] Christiansen G, Fastner J, Erhard M, Borner T, Dittmann E (2003). Microcystin biosynthesis in *Planktothrix*: genes, evolution, and manipulation. J. Bacteriol.

[b7] Christiansen G, Molitor C, Philmus B, Kurmayer R (2008). Nontoxic strains of cyanobacteria are the result of major gene deletion events induced by a transposable element. Mol. Biol. Evol.

[b8] Davis TW, Berry DL, Boyer GL, Gobler CJ (2009). The effects of temperature and nutrients on the growth and dynamics of toxic and non-toxic strains of *Microcystis* during cyanobacteria blooms. Harmful Algae.

[b10] Falconer IR (1991). Tumor promotion and liver injury caused by oral consumption of cyanobacteria. Environ. Toxicol. Water Qual.

[b11] Fortin N, Aranda-Rodriguez R, Jing H, Pick F, Bird D, Greer CW (2010). Detection of microcystin-producing cyanobacteria in Missisquoi Bay, Quebec, Canada, using quantitative PCR. Appl. Environ. Microbiol.

[b12] Gadkar VJ, Filion M, Filion M (2012). Studying microbial gene expression in complex environmental matrices using quantitative real-time polymerase chain reaction. Quantitative real-time PCR in applied microbiology.

[b13] Gobler CJ, Davis TW, Coyne KJ, Boyer GL (2007). Interactive influences of nutrient loading, zooplankton grazing, and microcystin synthetase gene expression on cyanobacterial bloom dynamics in a eutrophic New York lake. Harmful Algae.

[b14] Grosse Y, Baan R, Straif K, Secretan B, El Ghissassi F, Cogliano V (2006). Carcinogenicity of nitrate, nitrite, and cyanobacterial peptide toxins. Lancet Oncol.

[b15] Imamura S, Tanaka K, Shirai M, Asayama M (2006). Growth phase-dependent activation of nitrogen-related genes by a control network of group 1 and group 2 *σ* factors in a cyanobacterium. J. Biol. Chem.

[b16] Ito E, Kondo F, Terao K, Harada K-I (1997). Neoplastic nodular formation in mouse liver induced by repeated intraperitoneal injections of microcystin-LR. Toxicon.

[b17] Jacquet S, Briand J-F, Leboulanger C, Avois-Jacquet C, Oberhaus L, Tassin B (2005). The proliferation of the toxic cyanobacterium *Planktothrix rubescens* following restoration of the largest natural French lake (Lac du Bourget). Harmful Algae.

[b18] Jochimsen EM, Carmichael WW, An J, Cardo DM, Cookson ST, Holmes CEM (1998). Liver failure and death after exposure to microcystins at a hemodialysis center in Brazil. N. Engl. J. Med.

[b19] Kaebernick M, Neilan BA, Börner T, Dittmann E (2000). Light and the transcriptional response of the microcystin biosynthesis gene cluster. Appl. Environ. Microbiol.

[b20] Kaebernick M, Rohrlack T, Christoffersen K, Neilan BA (2001). A spontaneous mutant of microcystin biosynthesis: genetic characterization and effect on *Daphnia*. Environ. Microbiol.

[b21] Kaebernick M, Dittmann E, Börner T, Neilan BA (2002). Multiple alternate transcripts direct the biosynthesis of microcystin, a cyanobacterial. Appl. Environ. Microbiol.

[b22] Kardinaal WEA, Tonk L, Janse I, Hol S, Slot P, Huisman J (2007). Competition for light between toxic and nontoxic strains of the harmful cyanobacterium *Microcystis*. Appl. Environ. Microbiol.

[b23] Krüger T, Hölzel N, Luckas B (2012). Influence of cultivation parameters on growth and microcystin production of *Microcystis aeruginosa* (Cyanophyceae) isolated from Lake Chao (China). Microb. Ecol.

[b24] Kurmayer R, Kutzenberger T (2003). Application of real-time PCR for quantification of microcystin genotypes in a population of the toxic cyanobacterium *Microcystis* sp. Appl. Environ. Microbiol.

[b25] Kurmayer R, Dittmann E, Fastner J, Chorus I (2002). Diversity of microcystin genes within a population of the toxic cyanobacterium *Microcystis* spp. in Lake Wannsee (Berlin, Germany). Microb. Ecol.

[b27] Leblanc Renaud S, Pick FR, Fortin N (2011). Effect of light intensity on the relative dominance of toxigenic and nontoxigenic strains *of Microcystis aeruginosa*. Appl. Environ. Microbiol.

[b28] Lyck S (2004). Simultaneous changes in cell quotas of microcystin, chlorophyll a, protein and carbohydrate during different growth phases of a batch culture experiment with Microcystis aeruginosa. J. Plankton Res.

[b29] Mendes E, Mello M, Soares MCS, Roland F, Lürling M (2012). Growth inhibition and colony formation in the cyanobacterium Microcystis aeruginosa induced by the cyanobacterium Cylindrospermopsis raciborskii. J. Plankton Res.

[b30] Metcalf JS, Hyenstrand P, Beattie KA, Codd GA (2000). Effects of physicochemical variables and cyanobacterial extracts on the immunoassay of microcystin-LR by two ELISA kits. J. Appl. Microbiol.

[b31] Mikalsen B, Boison G, Skulberg OM, Fastner J, Davies W, Gabrielsen TM (2003). Natural variation in the microcystin synthetase operon mcyABC and impact on microcystin production in *Microcystis* Strains. J. Bacteriol.

[b32] Misson B, Sabart M, Amblard C, Latour D (2012). Benthic survival of *Microcystis*: long-term viability and ability to transcribe microcystin genes. Harmful Algae.

[b33] Ngwa F, Madramootoo C, Jabaji S (2012). Monitoring toxigenic Microcystis strains in the Missisquoi bay, Quebec, by PCR targeting multiple toxic gene loci. Environ. Toxicol.

[b34] Ngwa FF, Madramootoo CA, Jabaji S (2013). Development and application of a multiplex qPCR technique to detect multiple microcystin-producing cyanobacterial genera in a Canadian freshwater lake. J. Appl. Phycol.

[b35] Nishiwaki-Matsushima R, Ohta T, Nishiwaki S, Suganuma M, Kohyama K, Ishikawa T (1992). Liver tumor promotion by the cyanobacterial cyclic peptide toxin microcystin-LR. J. Cancer Res. Clin. Oncol.

[b36] Nishizawa T, Ueda A, Asayama M, Fujii K, Harada K-I, Ochi K (2000). Polyketide synthase gene coupled to the peptide synthetase module involved in the biosynthesis of the cyclic heptapeptide microcystin. J. Biochem.

[b37] Orr PT, Jones GJ (1998). Relationship between microcystin production and cell division rates in nitrogen-limited Microcystis aeruginosa cultures. Limnol. Oceanogr.

[b38] Ostermaier V, Kurmayer R (2009). Distribution and abundance of nontoxic mutants of cyanobacteria in lakes of the Alps. Microb. Ecol.

[b39] Philmus B, Christiansen G, Yoshida WY, Hemscheidt TK (2008). Post-translational modification in microviridin biosynthesis. Chem. BioChem.

[b40] Rantala A, Rajaniemi-Wacklin P, Lyra C, Lepisto L, Rintala J, Mankiewiez-Boczek J (2006). Detection of microcystin-producing cyanobacteria in Finnish lakes with genus-specific microcystin synthetase gene E (*mcyE*) PCR and associations with environmental factors. Appl. Environ. Microbiol.

[b41] Rapala J, Sivonen K, Lyra C, Niemela SI (1997). Variation of microcystins, cyanobacterial hepatotoxins, in Anabaena spp. as a function of growth stimuli. Appl. Environ. Microbiol.

[b42] Rivasseau C, Racaud P, Deguin A, Hennion M-C (1999). Evaluation of an ELISA kit for the monitoring of microcystins (Cyanobacterial Toxins) in water and algae environmental samples. Environ. Sci. Technol.

[b43] Rohrlack T, Christoffersen K, Kaebernick M, Neilan BA (2004). Cyanobacterial protease inhibitor microviridin J causes a lethal molting disruption in *Daphnia pulicaria*. Appl. Environ. Microbiol.

[b44] Rosen S, Skaletsky H (2000). Primer3 on the WWW for general users and for biologist programmers. Methods Mol. Biol.

[b45] Rouhiainen L, Vakkilainen T, Siemer BL, Buikema W, Haselkorn R, Sivonen K (2004). Genes coding for hepatotoxic heptapeptides (microcystins) in the cyanobacterium *Anabaena* strain 90. Appl. Environ. Microbiol.

[b46] Rueckert A, Cary SC (2009). Use of an armored RNA standard to measure microcystin synthetase E gene expression in toxic *Microcystis* sp. by reverse transcription QPCR. Limnol. Oceanogr.

[b47] Sartory DP, Grobbelaar JU (1984). Extraction of chlorophyll a from freshwater phytoplankton for spectrophotometric analysis. Hydrobiologia.

[b48] Schatz D, Keren Y, Hadas O, Carmeli S, Sukenik A, Kaplan A (2005). Ecological implications of the emergence of non-toxic subcultures from toxic *Microcystis* strains. Environ. Microbiol.

[b49] Schatz D, Keren Y, Vardi A, Sukenik A, Carmeli S, Börner T (2007). Towards clarification of the biological role of microcystins, a family of cyanobacterial toxins. Environ. Microbiol.

[b50] Sevilla E, Martin-Luna B, Vela L, Bes MT, Fillat MF, Peleato ML (2008). Iron availability affects *mcyD* expression and microcystin-LR synthesis in *Microcystis aeruginosa* PCC7806. Environ. Microbiol.

[b51] Sharkey FH, Banat IM, Marchant R (2004). A rapid and effective method of extracting fully intact RNA from thermophilic geobacilli that is suitable for gene expression analysis. Extremophiles.

[b52] Singh DP, Tyagi MB, Kumar A, Thakur JK, Kumar A (2001). Antialgal activity of a hepatotoxin-producing cyanobacterium, Microcystis aeruginosa. World J. Microbiol. Biotechnol.

[b53] Sivonen K, Herrero A, Flores E, Börner T (2008). Bioactive compounds produced by cyanobacteria. The cyanobacteria: molecular biology, genomics and evolution.

[b54] Spoof L, Vesterkvist P, Lindholm T, Meriluoto J (2003). Screening for cyanobacterial hepatotoxins, microcystins and nodularin in environmental water samples by reversed-phase liquid chromatography–electrospray ionisation mass spectrometry. J. Chromatogr. A.

[b55] Suda S, Watanabe MM, Otsuka S, Mahakahant A, Yongmanitchai W, Nopartnaraporn N (2002). Taxonomic revision of water-bloom-forming species of oscillatorioid cyanobacteria. Int. J. Syst. Evol. Microbiol.

[b56] Sukenik A, Eshkol R, Livne A, Hadas O, Rom M, Tchernov D (2002). Inhibition of growth and photosynthesis of the dinoflagellate *Peridinium gatunense* by *Microcystis* sp. (cyanobacteria): a novel allelopathic mechanism. Limnol. Oceanogr.

[b57] Tillett D, Dittmann E, Erhard M, Von Döhren H, Börner T, Neilan B (2000). Structural organization of microcystin biosynthesis in *Microcystis aeruginosa* PCC7806: an integrated peptide–polyketide synthetase system. Chem. Biol.

[b58] Tillett D, Parker D, Neilan B (2001). Detection of toxigenicity by a probe for the microcystin synthetase A gene (*mcyA*) of the cyanobacterial genus *Microcystis*: comparison of toxicities with 16S rRNA and phycocyanin operon (phycocyanin intergenic spacer) phylogenies. Appl. Environ. Microbiol.

[b59] Tonk L, Visser PM, Christiansen G, Dittmann E, Snelder EOFM, Wiedner C (2005). The microcystin composition of the cyanobacterium *Planktothrix agardhii* changes toward a more toxic variant with increasing light intensity. Appl. Environ. Microbiol.

[b60] Ueno Y, Nagata S, Tsutsumi T, Hasegawa A, Watanabe MF, Park H-D (1996). Detection of microcystins, a blue-green algal hepatotoxin, in drinking water sampled in Haimen and Fusui, endemic areas of primary liver cancer in China, by highly sensitive immunoassay. Carcinogenesis.

[b61] Vaitomaa J, Rantala A, Halinen K, Rouhiainen L, Tallberg P, Mokelke L (2003). Quantitative real-time PCR for determination of microcystin synthetase E copy numbers for *Microcystis* and *Anabaena* in lakes. Appl. Environ. Microbiol.

[b62] Van Der Westhuizen AJ, Eloff JN (1983). Effect of culture age and pH of culture medium on the growth and toxicity of the blue-green alga *Microcystis aeruginosa*. Zeitschrift Pflanzenphysiol.

[b64] Vezie C, Brient L, Sivonen K, Bertru G, Lefeuvre JC, Salkinoja-Salonen M (1998). Variation of microcystin content of cyanobacterial blooms and isolated strains in Lake Grand-Lieu (France). Microb. Ecol.

[b65] Vézie C, Rapala J, Vaitomaa J, Seitsonen J, Sivonen K (2002). Effect of nitrogen and phosphorus on growth of toxic and nontoxic *Microcystis* strains and on intracellular microcystin concentrations. Microb. Ecol.

[b66] Via-Ordorika L, Fastner J, Kurmayer R, Hisbergues M, Dittmann E, Komarek J (2004). Distribution of microcystin-producing and non-microcystin-producing *Microcystis* sp. in European freshwater bodies: detection of microcystins and microcystin genes in individual colonies. Syst. Appl. Microbiol.

[b67] Voet D, Harris D, Fitzgerald P, Voet JG (2004). Prokaryotic mRNA have short lifetimes. Biochemistry.

[b68] Walsh CT, Gehring AM, Weinreb PH, Quadri LEN, Flugel RS (1997). Post-translational modification of polyketide and nonribosomal peptide synthases. Curr. Opin. Chem. Biol.

[b69] Watanabe MF, Oishi S (1985). Effects of environmental factors on toxicity of a cyanobacterium (*Microcystis aeruginosa*) under culture conditions. Appl. Environ. Microbiol.

[b70] Welker M, Von Döhren H (2006). Cyanobacterial peptides; Nature's own combinatorial biosynthesis. FEMS Microbiol. Rev.

[b71] Wood SA, Heath MW, Holland PT, Munday R, Mcgregor GB, Ryan KG (2010). Identification of a benthic microcystin-producing filamentous cyanobacterium (Oscillatoriales) associated with a dog poisoning in New Zealand. Toxicon.

[b72] Wood SA, Rueckert A, Hamilton DP, Cary SC, Dietrich DR (2011). Switching toxin production on and off: intermittent microcystin synthesis in a *Microcystis* bloom. Environ. Microbiol. Rep.

[b73] Xu H, Paerl HW, Qin B, Zhu G, Gao G (2010). Nitrogen and phosphorus inputs control phytoplankton growth in eutrophic Lake Taihu. China. Limnol. Oceanogr.

[b74] Yoshida M, Yoshida T, Takashima Y, Kondo R, Hiroishi S (2005). Genetic diversity of the toxic cyanobacterium Microcystis in Lake Mikata. Environ. Toxicol.

[b75] Yu S-Z (1995). Primary prevention of hepatocellular carcinoma. J. Gastroenterol. Hepatol.

[b76] Zhao S, Fernald RD (2005). Comprehensive algorithm for quantitative real-time polymerase chain reaction. J. Comput. Biol.

[b77] Zhou L, Yu H, Chen K (2002). Relationship between microcystin in drinking water and colorectal cancer. Biomed. Environ. Sci.

