# Supplementary figures and images for: Comparison of cyanobacterial microcystin synthetase (mcy) E gene transcript levels, mcy E gene copies, and biomass as indicators of microcystin risk under laboratory and field conditions
Source: Microbiologyopen. 2014 May 17;3(4):411–25. doi: 10.1002/mbo3.173 (PMC4287171; doi:10.1002/mbo3.173)

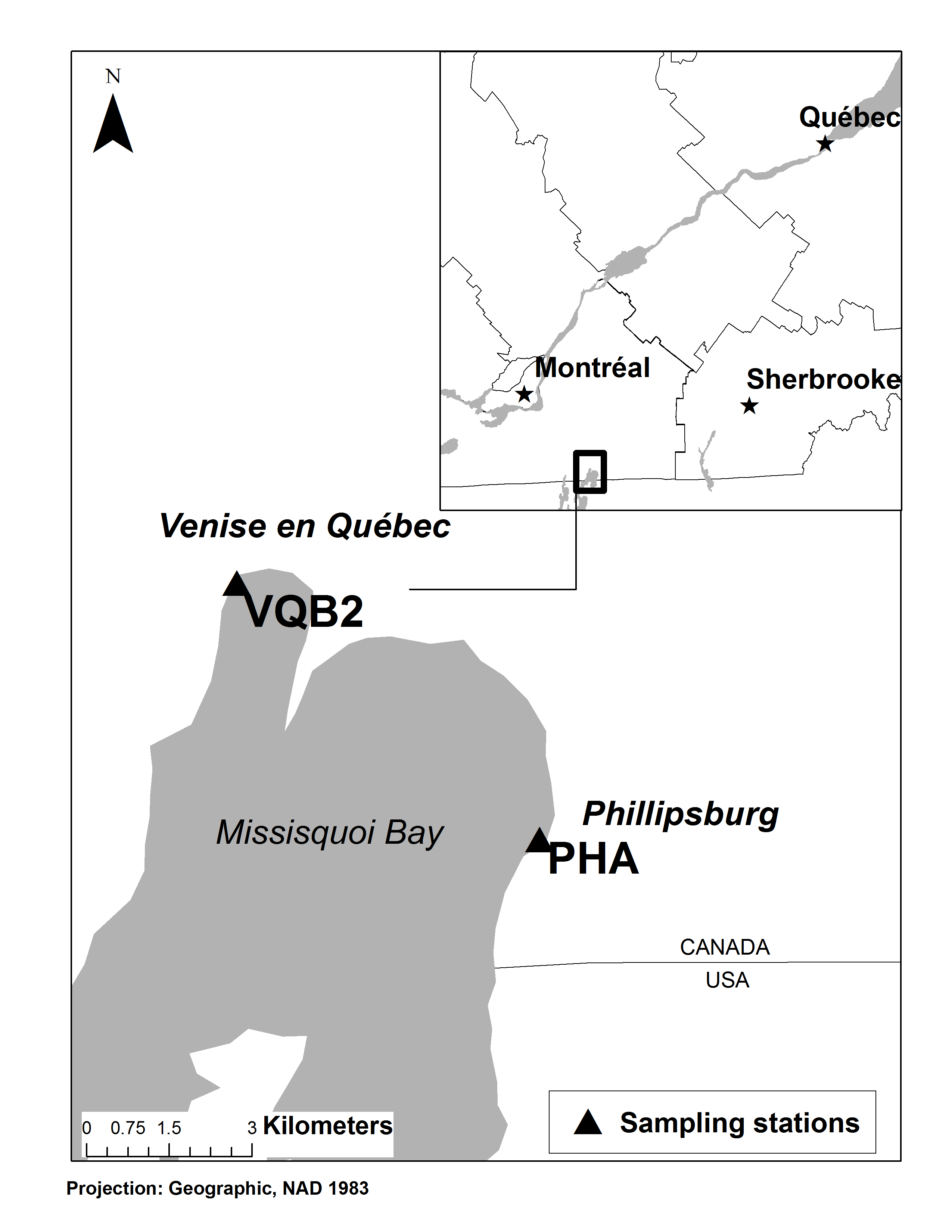

Supplement: Figure S1 — Location of the PH-A and VQ-B2 sampling stations in the Missisquoi Bay Quebec (Source: Ngwa et al. 2013). [file mbo30003-0411-sd1.tif]

## Slide 1
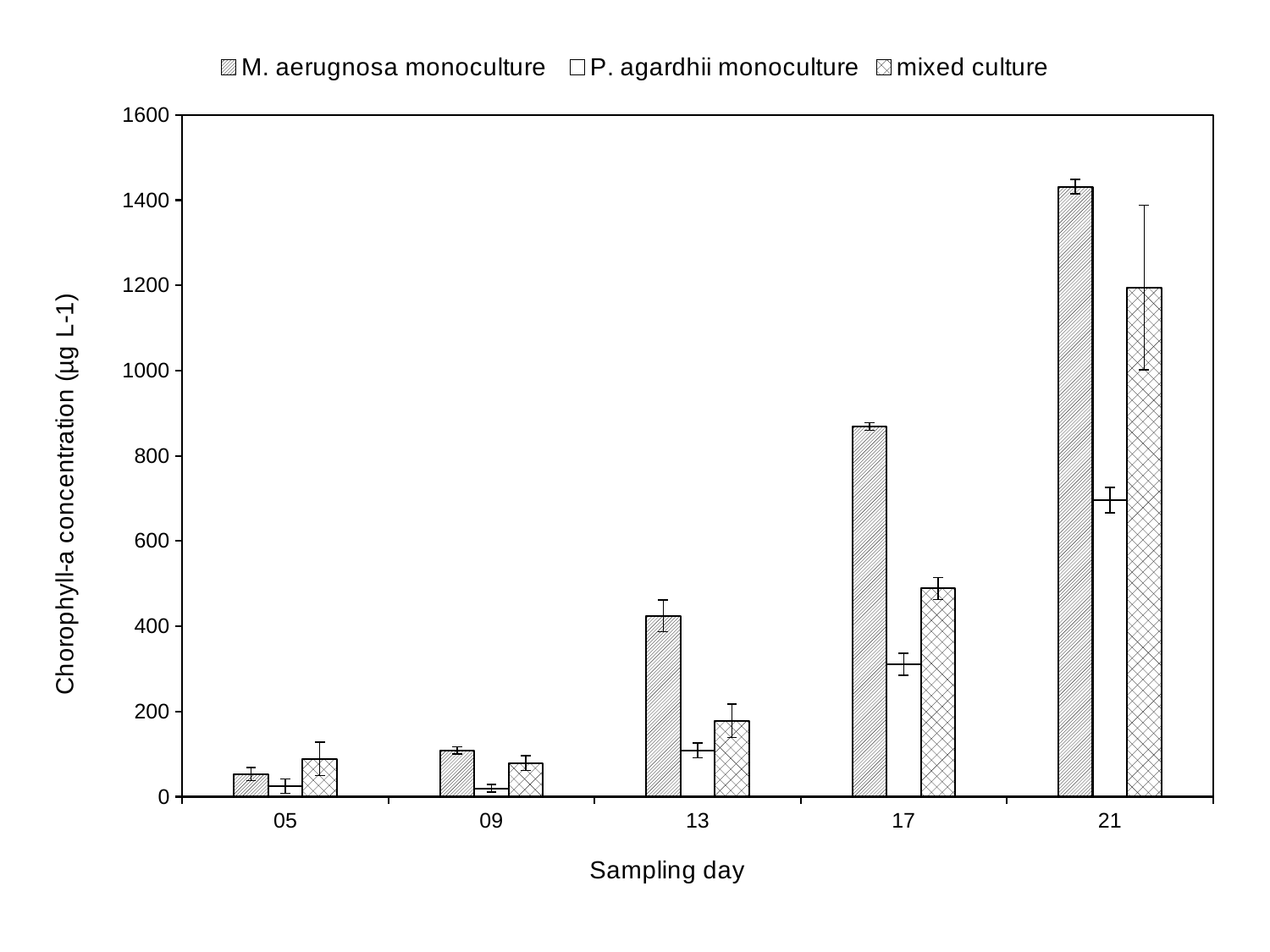

### Chart
| Category | | | |
|---|---|---|---|
| 41218 | 53.315999999999995 | 24.683333333333334 | 88.86 |
| 41222 | 108.60666666666667 | 19.746666666666666 | 78.98666666666666 |
| 41226 | 424.5533333333333 | 108.60666666666668 | 177.72000000000003 |
| 41230 | 868.8533333333335 | 311.01 | 488.73 |
| 41234 | 1431.6333333333332 | 696.07 | 1194.6733333333334 |

Supplement: Figure S2 — Changes in chlorophyll-a concentration in Microcystis aeruginosa and Planktothrix agardhii under laboratory conditions. [file mbo30003-0411-sd2.pptx]
