# Supplementary material for: Comparison of cyanobacterial microcystin synthetase (mcy) E gene transcript levels, mcy E gene copies, and biomass as indicators of microcystin risk under laboratory and field conditions
Source: Microbiologyopen. 2014 May 17;3(4):411–25. doi: 10.1002/mbo3.173 (PMC4287171; doi:10.1002/mbo3.173)
Supplement: Table S1 — F-statistics for type III tests of fixed effects. [file mbo30003-0411-sd3.docx]

Table S1: F-statistics for Type III Tests of Fixed Effects

|  | **Microcystin** | | **Cell counts** | | ***mcyE* copies** | | ***mcyE* expression** | |
| --- | --- | --- | --- | --- | --- | --- | --- | --- |
|  | *Microcystis* | *Planktothrix* | *Microcystis* | *Planktothrix* | *Microcystis* | *Planktothrix* | *Microcystis* | *Planktothrix* |
| Trt | 0.91^nss^ | 211.87** | 983.27*** | 0.99^nss^ | 84.24** | 46.92*** | 198.87*** | 446.93*** |
| day | 1110.93*** | 672.34*** | 2385.21*** | 50.60*** | 1504.29*** | 8.53** | 15.82*** | 99.58*** |
| Trt*day | 25.66*** | 13.04** | 104.98*** | 11.68** | 45.19*** | 3.44* | 42.22*** | 119.71*** |

*; p< 0.05, **; p<0.01; ***; p<0.0001, nss; not statistically significant
